# Current Advances in Specialised Niosomal Drug Delivery: Manufacture, Characterization and Drug Delivery Applications

**DOI:** 10.3390/ijms23179668

**Published:** 2022-08-26

**Authors:** Bwalya A. Witika, Kokoette E. Bassey, Patrick H. Demana, Xavier Siwe-Noundou, Madan S. Poka

**Affiliations:** Department of Pharmaceutical Sciences, School of Pharmacy, Sefako Makgatho Health Sciences University, Pretoria 0208, South Africa

**Keywords:** magnetic niosomes, immuno-niosomes, thermoresponsive niosomes, pH-sensitive niosomes, targeted drug delivery

## Abstract

Development of nanomaterials for drug delivery has received considerable attention due to their potential for achieving on-target delivery to the diseased area while the surrounding healthy tissue is spared. Safe and efficiently delivered payloads have always been a challenge in pharmaceutics. Niosomes are self-assembled vesicular nanocarriers formed by hydration of a non-ionic surfactant, cholesterol or other molecules that combine to form a versatile drug delivery system with a variety of applications ranging from topical delivery to targeted delivery. Niosomes have advantages similar to those of liposomes with regards to their ability to incorporate both hydrophilic and hydrophobic payloads. Moreover, niosomes have simple manufacturing methods, low production cost and exhibit extended stability, consequently overcoming the major drawbacks associated with liposomes. This review provides a comprehensive summary of niosomal research to date, including the types of niosomes and critical material attributes (CMA) and critical process parameters (CPP) of niosomes and their effects on the critical quality attributes (CQA) of the technology. Furthermore, physical characterisation techniques of niosomes are provided. The review then highlights recent applications of specialised niosomes in drug delivery. Finally, limitations and prospects for this technology are discussed.

## 1. Introduction

In recent times, there has been an increased amount of attention focused on the development of novel drug delivery systems (NDDS). Ideally, these NDDS should be able to fulfil two prerequisites: the capability of delivering payload at a rate directed by the needs of the patient consistently over the treatment duration, and focusing the delivery of the payload to the site of action at which it is required [1]. Presently, conventional dosage forms, inclusive of prolonged-release dosage forms, are unable to meet these requirements [1]. Novel drug delivery approaches attempt to sustain drug action at a predetermined rate or by maintaining a relatively constant, effective level of API in the body with minimisation of undesirable side effects in addition to localised drug action through the spatial placement of controlled-release systems adjacent to or in the diseased tissue and/or organ or targeted drug action through the use of carriers or chemical derivatisation to target a particular cell type [1].

Vesicular systems have become a useful approach for delivering payload to specific sites and improving outcomes. Lipid vesicles have found applications in immunology, membrane biology, diagnostics and genetic engineering research [2,3,4]. Vesicles can also be used in modelling biological membranes, and for the transport and targeting of API. 

Biological membranes are universal delimiting structures that surround and compartmentalise all cells and organelles. The bilayer arrangement of lipids is the only organisational feature common to all biological membranes. Experimental models provide insight into the motional dynamics and static structures of some isolated compartments of biological membranes and lipid vesicles, and are just one type of the experimental models of biomembranes. Despite being developed for basic research, many technological innovations have arisen from applications of these models, and lipid vesicles have successfully evolved as suitable vehicles for controlled delivery [1]. 

Conventional chemotherapy for the treatment of intracellular infections is not effective due to limited cellular permeation of the payload as well as significant “off-target” accumulation of the API. This may be overcome by the use of vesicular drug delivery systems. Payload encapsulation in a vesicular structure has potential for availability in systemic circulation and to reduce “off-target” accumulation [5]. Phagocytic uptake following systemic delivery of loaded vesicular systems provides an efficient method of delivery to the site of infection, which could result in a reduction in toxicity with few, if any, adverse effects.

Niosomes are microscopic lamellar structures that are similar to liposomes but are composed of non-ionic surfactants rather than phospholipids [6,7]. The amphiphiles involved when producing niosomes include Spans^®^ [8,9,10,11] and/or Tweens^®^ [11,12] and/or ethoxylated alcohols and/or sucrose esters that are stabilized by the addition of cholesterol and small amounts of ionic materials such as diacetyl phosphate or chitosan [13,14,15,16].

**Figure 1:**
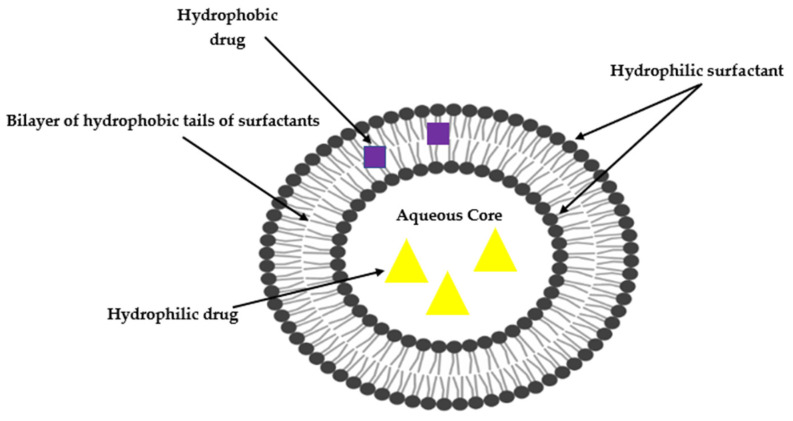
Schematic representation of a typical bilayered lipid vesicle.

Niosomes, like other bilayered vesicular drug delivery systems, can entrap and shuttle both lipophilic and hydrophilic payloads in either the bilayer membrane or aqueous layer, respectively [17,18]. Studies have suggested that niosomes are more stable than liposomes [19], and the presence of non-ionic surfactants facilitates prolonged circulation of the carriers, potentially enhancing therapy and targeting delivery to specific organs such as the brain, liver and tumours [14,20]. A schematic representation of a niosome is depicted in Figure 1.

Niosomes offer many advantages over conventional drug delivery systems in terms of flexibility for drug delivery and the capability to entrap hydrophilic and hydrophobic drugs. Niosomes entrap solutes and API of different solubility, delivering them via many different routes of administration: oral, pulmonary, ocular, parenteral and topical [21]. The surfaces of the vesicle structure can be functionalised for targeted delivery, and therapeutic performance may be enhanced due to reduced clearance from the biological system. The surfactants used in the manufacture of niosomes are biodegradable, biocompatible and nonimmunogenic, making them ideal for human use. Niosomes are osmotically active and stable, and may enhance the stability of the payload [22,23,24,25].

Niosomes exhibit advantages over other vesicle-based drug delivery systems, and their ease of manufacture is convenient for the easy entrapment of API when compared to pharmacosomes that require complex covalent bonding approaches to entrap compounds, which makes it difficult to incorporate all drugs. Niosomes exhibit other advantages over liposomes with respect to stability and overall circulation half-life. The presence of phospholipids results in liposomes being phagocytosed more rapidly than niosomes that include surfactants. Phospholipids used in the manufacture of liposomes are also expensive and have a propensity to degrade [20,26].

This review introduces the design and fabrication of niosomes as drug delivery systems. Furthermore, the paper discusses the characterisations associated with niosomes before introducing the different types of specialised niosomes to highlight how niosomes are tuneable as drug carriers, resulting in better performance for different diseases and conditions.

## 2. Preparation of Niosomes

Niosomes, like liposomes, can be categorized broadly according to their lamellarity. The bilayered vesicles may be Small Unilamellar Vesicles (SUV), Large Unilamellar Vesicles (LUV) or Multi-Lamellar Vesicles (MLV). Different sizes of niosomes may be necessary for the optimisation of specific delivery requirements, and several different approaches are used to produce these nanocarrier systems. 

Niosomes can be prepared by different methods depending on the desired size of vesicle required, size distribution, number of bilayers, entrapment efficiency of the aqueous phase and permeability of the vesicle membranes required. In general, niosomes are manufactured by hydration of a mixture of surfactant and lipid at an elevated temperature, usually >60 °C, using an aqueous medium followed by an optional size reduction process and ultimately the formation of a colloidal dispersion [20,27].

### 2.1. Preparation of Multilamellar Vesicles (MLV)

#### 2.1.1. Thin-Film Hydration

Thin-film hydration (TFH), frequently termed the hand-shaking method, involves dissolving a surfactant and cholesterol and hydrophobic payload in a volatile organic solvent, or mixtures thereof, such as chloroform, diethyl ether, ethanol or methanol. The organic solvent is then evaporated using a rotary evaporator, which results in the formation of a thin layer of solids being deposited on the wall of a round-bottom flask [9,10,11,28].

The dried thin layer is subsequently hydrated with an aqueous phase that may contain the hydrophilic drug. The hydration step is conducted at temperatures above the glass transition temperature (T_c_) of the surfactant, usually >60 °C, with gentle agitation [5,29,30]. While forceful hydration of the surfactant film produces multilamellar niosomes of heterogeneous size, gentle hydration leads to the formation of giant unilamellar vesicles (GUV).

Generally, the use of the TFH requires the use of sonication or extrusion to produce small monodisperse niosomes [31]. Most frequently, this size reduction is by use of probe or bath sonication and results in the formation of SUV [31]. Probe sonication can produce local heat, which can affect the chemical stability of surfactants and API while also raising the possibility of contamination from the probe. Bath sonication is an effective alternative due to the possibility of controlling sonication parameters while producing monodisperse niosomes [31].

Another technique used for size reduction involves consecutive extrusion of the preparation through polycarbonate filters. Effective size reduction using this method is related to the number of extrusion cycles and the pore size of filter membranes. The primary challenge of TFH is that the entrapment of an aqueous core is limited, often resulting in poor encapsulation efficiency of hydrophilic drugs [20,29,32]. 

Colchicine [33], insulin [34], nevirapine [9,11,35], tenofovir disoproxil fumarate [36] and doxorubicin [37] have successfully been encapsulated using this approach. This is further evidence of the versatility of the technique. 

#### 2.1.2. Transmembrane pH Gradient Drug Uptake

This approach is highly convenient for hydrophobic payload that is ionizable. In this approach, hydrophobic payload, surfactant and cholesterol are dissolved in chloroform. The solvent is then evaporated under reduced pressure to produce a thin film on the wall of a round-bottom flask. The film is then hydrated with 300 mM citric acid at pH 4.0 with the aid of a vortex mixer. The multilamellar vesicles formed are subsequently frozen and thawed three times, followed by sonication. An aqueous solution containing the drug is then added to the niosome suspension and vortexed. The pH of the sample is then raised to between 7.0 and 7.2 with a 1 M disodium phosphate solution, after which the mixture is heated at 60 °C for 10 min to produce multilamellar vesicles [38].

The neutral exterior of the vesicles results in the presence of protonated and unprotonated forms of the payload. The unprotonated form of the payload is permeable and tends to permeate across the niosome membrane. The API then becomes protonated in the acidic contents of the vesicle and remains trapped in the vesicle. Diffusion continues until the API concentration is in equilibrium on the interior and exterior of the vesicle membrane [39].

### 2.2. Preparation of Small Unilamellar Vesicle (SUV)

#### 2.2.1. Microfluidics 

Microfluidic methods include all the novel preparation techniques that use micro-channels of diameter between 5 µm and 500 µm to control material flow. The fluidized stream of lipids in ethanol or isopropanol moves through precisely defined microchannels and interacts with an aqueous stream at ultra-high velocities within an interaction chamber. Axial mixing of the two phases occurs continuously, and niosomes are formed by local diffusion of surfactant molecules that self-assemble in contact with the aqueous phase. Niosomes formed with this technology exhibit a high degree of reproducibility and uniformity, with formation of vesicles of small size of <150 nm and very low PDI < 0.200 [40,41].

#### 2.2.2. Sonication

When using sonication, an aqueous phase containing a drug is added to a mixture of surfactant and cholesterol in a scintillation vial [1,29]. The mixture is then homogenised using a sonic probe at 60 °C for up to 3 min. The vesicles that are produced are small and uniform in size. Sonication is conducted at temperatures above the phase transition temperature and for >20 min.

### 2.3. Preparation of Large Unilamellar Vesicles

#### 2.3.1. Reversed-Phase Evaporation (REV)

The REV approach for niosome preparation is mainly desired in order to encapsulate hydrophilic payload, as it ensures a more highly aqueous core than the film hydration method. The REV approach requires mixing cholesterol and surfactant in a mixture of ether and chloroform. An aqueous solution containing payload is added, and the two phases that form are sonicated for 5 min at 4–5 °C. The clear gel that is formed is further sonicated following the addition of approximately 10 mL of aqueous phase, after which the organic phase is removed under low pressure at ~40 °C using a rotary evaporator. The resulting viscous suspension is diluted with an aqueous phase and heated in a water bath at temperatures above the T_c_ for 10 min to yield niosomes [29,42].

The main drawback of this technique is the possibility of the presence of residual solvent, which may result in unintended biological effects.

#### 2.3.2. Ether Injection

The ether injection method is based on the slow injection of niosome components in ether, ethanol or isopropyl alcohol through a 14-gauge needle into a preheated aqueous phase maintained at 60 °C at a rate of approximately 0.25 mL/min [29,43,44]. LUV are formed due to slow vaporisation of the solvent, resulting in an ether gradient extending towards the aqueous–non-aqueous liquid interface, and the former may be the reason that a bilayer structure forms [29,43]. Niosomes manufactured during injection are heterogeneous in size and retain minimal amounts of ether. In some instances, ethanol forms an azeotropic mixture with water; this and exposure of the payload to a high temperature are the main drawbacks of this approach [26,29,43,45]. 

### 2.4. Miscellaneous Techniques

#### 2.4.1. Emulsion Formation 

The initial step in this technique is the formation of an oil-in-water (o/w) emulsion from an organic solution of surfactant, cholesterol and an aqueous solution of the payload [33]. The organic solvent is then evaporated, resulting in the formation of niosomes dispersed in the aqueous phase.

#### 2.4.2. Niosome Manufacture Using Micelles

Niosomes may also be developed from a mixed micelle solution with the aid of enzymes. A mixed micellar solution of hexadecyl diglycerol (C_16_G_2_), polyoxyethylene cholesteryl sebacetate diester (PCSD) and dicalcium hydrogen phosphate converts to a niosome dispersion when incubated with esterase enzyme substrates. The PCSD is cleaved by the esterase to yield polyoxyethylene, sebacic acid and cholesterol, and cholesterol in combination with C_16_G_2_ and DCP then yields C_16_G_2_ niosomes [38,46].

#### 2.4.3. Lipid Injection

The lipid injection approach does not make use of expensive organic phases and is considered green. A mixture of lipids and surfactant are initially melted then subsequently injected into a highly agitated hot aqueous solution of payload [1,31,47].

#### 2.4.4. Niosome Manufacture Using Polyoxyethylene Alkyl Ether

When using polyoxyethylene alkyl ether and cholesterol, the size and number of bilayers in vesicles can be changed using homogenisation [34]. Temperatures > 60 °C transform small ULV into large MLV that are >1 μm in diameter, whereas vigorous shaking at 22 °C exerts the converse effect, and MLV transform to LUV. The transformation from unilamellar to multilamellar vesicles at higher temperatures may be attributed to the characteristics of the polyoxyethylene alkyl ether (ester) surfactant since it is known that polyethylene glycol (PEG) and water mix at higher temperatures due to breakdown of hydrogen bonds between the water and PEG moieties. This allows for the vesicles to absorb more water and hydrate, further increasing the particle size of the vesicles [34].

**Figure 2:**
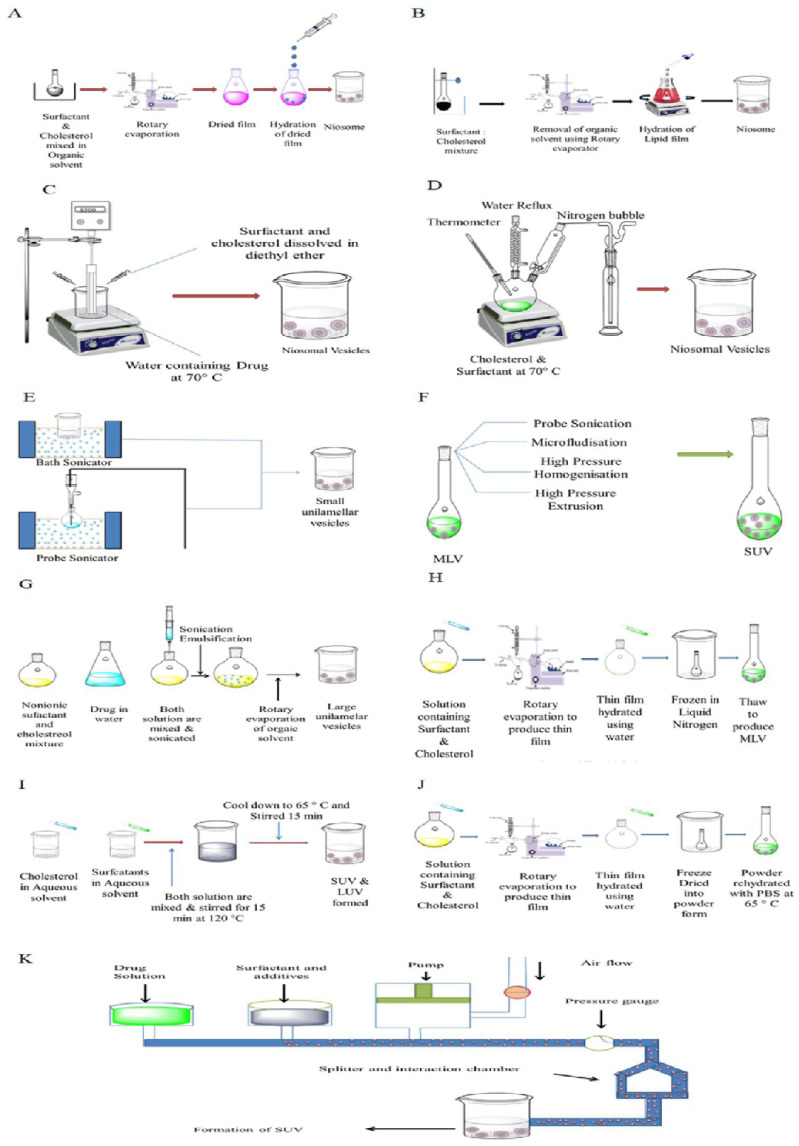
Schematic representation of different techniques for preparation of niosomes: (**A**) thin-film hydration, (**B**) hand-shaking, (**C**) ether injection, (**D**) bubble, (**E**) sonication, (**F**) proniosome technology, (**G**) reverse phase evaporation, (**H**) freeze-and-thaw, (**I**) heating, (**J**) dehydration and rehydration and (**K**) microfludics. (Reproduced from [48] with permission from Elsevier and in accordance with the Creative Commons Attribution License).

The techniques used to manufacture niosomes are summarised in Figure 2.

## 3. Characterisations of Niosomes

The physicochemical stability and biological fate of niosomes can be estimated by determining specific properties. In general, niosomes are characterized using particle size (PS), Zeta potential (ZP), polydispersity index (PDI), particle shape, surface morphology, lamellarity, encapsulation efficiency, phase behaviour/polymorphism, in vitro drug release, and in vivo performance.

### 3.1. Particle Size (PS) and Polydispersity Index (PDI)

Niosomes are assumed to be spherical, and their mean diameter can be determined using laser light scattering [49]. TEM can also be used to determine the diameter of niosomes [5,50,51]. Molecular sieve chromatography, ultracentrifugation, photon correlation microscopy and optical microscopy have also been used to measure the size of niosomes [52,53].

PS is an indicator of the ability of niosomes to penetrate biological barriers such as the blood–brain barrier (BBB). Small particles have a tendency to cross the BBB, which is important for targeted drug delivery to the brain or for phagocytosis by macrophages in cases of diseases such as tuberculosis and HIV/AIDS [54,55].

The PDI of a formulation is a measure of the particle size distribution of the niosomes in the formulation, with a small PDI generally preferred in order to permit accurate estimation of particle behaviour [45].

The PDI of a dispersion is measured by using dynamic light scattering (DLS), photon correlation spectroscopy (PCS) [56] or quasi-elastic light scattering [57], and is the most frequently used technique for particle size and polydispersity analysis. DLS is used to monitor the Brownian motion of the particles as a consequence of the bombardment by solvent molecules scattering the applied light. The instrument estimates the rate, time-dependent fluctuations and intensity of scattered light, which are related to the rate of diffusivity of dispersed particles. Smaller particles diffuse faster than larger particles, and the difference between the refractive index of the solvent and the particles governs the degree of light scattering.

The average particle size of a dispersion is automatically calculated by estimating the amount of light that has been scattered, and this approach is simple, rapid and reliable for routine use. DLS has an advantage when undertaking particle size analysis on native niosomal preparations as it can be used to assess a wide size range of between a few nanometres to several micrometres. Nevertheless, this technique shows limitations. DLS does not differentiate individual particles from particle aggregates, and the effect of particle solvation cannot be removed from the data. The size measured by DLS is considered a hydrodynamic diameter, and particle size analysis using DLS is highly sensitive to particulate contamination [45,58].

### 3.2. Zeta Potential (ZP)

The ZP of niosomal dispersions corresponds to the sum of surface charges, which is dependent on the components used in the formulation. Analysis of the surface charge of the niosomes is vital to estimate the stability of the technology long-term and the in vivo performance and the biological fate of the carriers. Niosomes with a large negative or positive charge exhibit negligible aggregation in a dispersion compared to neutral and slightly charged niosomes. This is attributed to the lack of electrostatic repulsive forces in neutral dispersions.

In the measurement of ZP, DLS is used to estimate the changes in intensity of the scattered light due to the mobility of niosomes as a result of the impact of the electric field applied on particle charges. The charge on the surface of niosomes governs mobility, which changes the intensity of the scattered light [37,45,59]

### 3.3. Encapsulation Efficiency (EE)

Encapsulation efficiency (EE) is a measure of the extent a payload has been successfully encapsulated by the niosome. It is cardinal to quantitate the EE, as it dictates the dose of the formulation to eventually be administered and has an impact on the payload release rate [31].

Following the manufacture of a niosomal dispersion, any API that has not been encapsulated is removed by centrifugation of dialysis. Subsequently, the payload of the niosomes is estimated after disrupting the vesicle with 50% v/v n-propanol or 0.1% w/v Triton X-100 solution. The resultant is analysed using a validated assay procedure for the payload [24]. The entrapment efficiency is then calculated using Equation (1) [29,60].

(1) $$EE=\frac{Amount\;of\;API\;entrapped\;}{Total\;amount\;of\;API}\times 100$$

### 3.4. Phase Behaviour

Polymorphism is an important parameter to be considered when determining the stability of a system and attempting to predict API release. Crystalline niosomes are more stable and exhibit sustained drug release profiles when compared to amorphous niosomes [61]. 

The most frequently used techniques for investigating the phase behaviour of niosomes include thermal analysis techniques, such as thermogravimetric analysis (TGA) and differential scanning calorimetry (DSC), and crystallographic analysis, such as X-ray diffraction (XRD). By investigating the thermal behaviour and crystallinity of such a system using these techniques, valuable complementary information can be generated for the characterisation and quality control of niosomal formulations [25,26].

### 3.5. In Vitro Drug Release

In vitro payload release behaviour from niosomes is a fundamental parameter to be influenced and includes drug concentration, hydration volume and rigidity and state of the bilayer.

The release of drug molecules from niosomes is generally studied using dialysis membrane techniques [9,10,11,12,62]. Purified niosomal suspension is inserted into a dialysis bag then sealed off at both ends and placed in a vessel such as a beaker of simulated bodily fluids or phosphate buffered saline and maintained at a temperature of 37 °C with agitation. Samples are taken at predetermined time intervals and replaced with the same amount of fresh medium [9,10,11,12,62]. These samples are then analyzed using appropriate quantitation techniques to determine the concentration of payload released over time [9,10,11,12,62]. Other alternative methods using Franz diffusion cells have been reported and follow similar general procedures.

### 3.6. Surface Elemental Composition

The surface composition of niosomes is an important parameter in determining whether the payload is adsorbed on the niosome surface, which could be a predictor of burst release and encapsulation efficiency. 

Elemental dispersive spectroscopy (EDX), a technique coupled with SEM, has widely been used for this purpose. Though not widely used in niosomes, the technique has been used in liposomes with considerable success to determine whether successful drug loading occurred [63,64].

X-ray photoelectron spectroscopy (XPS) is another technique that could be useful for the determination of the surface elemental composition of niosomes. It has not been reported in use in niosomes, but its use in liposomes has been reported and can be extrapolated to niosomes [65].

### 3.7. Bilayer Formation

The assembly of non-ionic surfactants to form bilayered vesicles is characterised by the formation of an X-cross using light polarisation microscopy [66]. The use of stained transmission electron microscopy (TEM) could also be used to establish the formation of bilayers [49,67].

### 3.8. Number of Lamellae

The number of lamellae is established using nuclear magnetic resonance (NMR) spectroscopy, small angle X-ray scattering and TEM [20,49,67].

### 3.9. Membrane Rigidity

Membrane rigidity may be measured by means of the mobility of a fluorescence probe as a function of temperature [66]. The rigidity of the niosomal bilayer has a direct impact on in vitro and in vivo performance with regard to drug release.

## 4. Factors Affecting the Critical Quality Attributes (CQA) of Niosomes

Several factors affect the physicochemical properties of niosomes and are discussed in detail herein. Properties such as PS, EE, particle shape, payload, release kinetics and ZP all impact the performance and therapeutic capability of niosomes.

### 4.1. Choice of Surfactant

Surfactants used for the manufacture of niosomes must have a hydrophilic or polar head and a lipophilic hydrophobic tail. The hydrophobic tail usually consists of one or two alkyl or perfluoroalkyl functional groups or a single steroidal group [20]. The ether-like surfactants with single alkyl chains are more toxic than the corresponding dialkyl ether compounds. Ester-containing surfactants are chemically less stable than ether-containing surfactants, and the latter is more toxic due to ester-linked surfactant degradation by esterase enzymes to form triglycerides and fatty acids in vivo [32]. Surfactants with alkyl chain lengths between C_12_ and C_18_ are suitable for the manufacture of niosomes [68]. 

The hydrophilic/lipophilic balance (HLB) of non-ionic surfactants is a reflection of the proportion weight % of hydrophilic and lipophilic groups in a molecule [69]. The HLB value of a surfactant can be indicative of the vesicle-forming ability of the material. Surfactants such as Tween^®^ 20, 60 and 80 with HLB values between 14 and 17 are not able to form vesicles without the addition of lipids [70]. However, surfactants such as Span^®^ with HLB values < 8 have the ability to make vesicles [20,71].

The choice of the surfactant has been demonstrated to have a direct impact on the PS, PDI and, to a lesser extent, on the ZP. Surfactants with a larger HLB generally exhibit a PS that is usually accompanied by a larger PDI [10,11].

### 4.2. Critical Packing Parameters (CPP)

**Figure 3:**
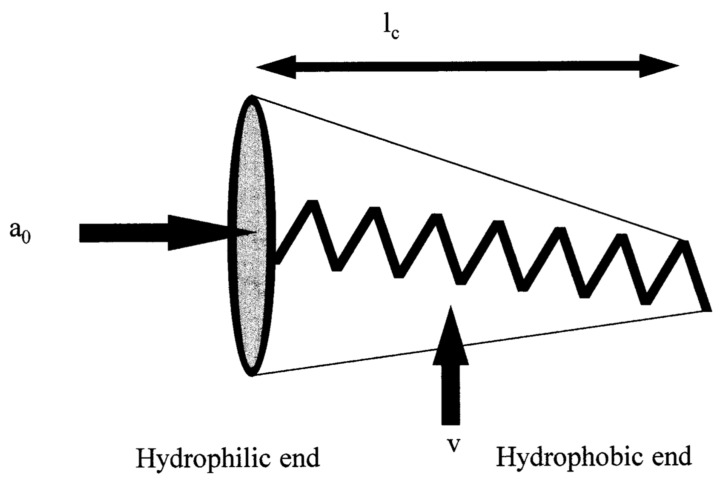
Schematic representation of an amphiphile: a_0_, hydrophilic head group area: v, hydrophobic chain volume; and l_c_, hydrophobic chain length. (Reproduced from [20] with permission from Elsevier, Amsterdam.).

In addition to the overall surfactant choice and subsequent HLB number, prediction of the surfactant system’s formation of vesicles requires an understanding of the chemical structure and various other factors regarding the surfactants. CPP is a dimensionless scale of surfactants and is defined by Equation (2) [72] and depicted visually in Figure 3 [20].

(2) $$\mathrm{CPP}=\frac{v}{l_{c}a_{0}}$$

where v is the hydrophobic group volume, l_c_ is the critical hydrophobic group length and a_0_ is the area of the hydrophilic head group.

Predicting the type of niosome formed can be achieved by understanding an amphiphiles ability to form spherical micelles, non-spherical micelles, bilayer vesicles or inverted micelles, which are associated with CPP < ⅓, ⅓ < CPP < ½, ½ < CPP < 1 or CPP ≥ 1, respectively [19,34,73].

### 4.3. Nature of Payload

The physicochemical properties of an encapsulated drug may affect the charge and rigidity of the bilayers of a niosome. The payload may interact with the polar head groups of the surfactant used and develop a charge that repels the surfactant bilayers, thereby increasing the PS [20,43,74]. Aggregation of vesicles can be prevented by establishing a charge on the surface of the bilayer. 

Payload can generally be classified in terms of solubility as hydrophilic, hydrophobic or amphiphilic [41]. The nature of the API affects the EE, stability and leakage from niosomes [75]. Hydrophobic and large macromolecule compounds exhibit superior EE compared to low MW and hydrophilic compounds [76].

### 4.4. Cholesterol Content

The inclusion of cholesterol in niosomes increases the hydrodynamic diameter and entrapment efficiency of the technology. In general, cholesterol increases the ordered nature of the bilayers in the liquid state and conversely decreases the order of the bilayers in the gel state. When high concentrations of cholesterol are used, the gel state transforms to produce an ordered liquid phase. An increase in the cholesterol content within the bilayers results in a decrease in the rate of release of encapsulated materials as the rigidity of the bilayers is increased. The presence of charges tends to result in an increase in the interlamellar distance between successive bilayers of MLV structures and leads to a greater overall volume for entrapment [68].

### 4.5. Charge Inducers

As a consequence of their non-ionic nature, niosomes usually require additives to impart surface charge, which subsequently affects ZP and, by virtue of a knock-on effect, dispersion stability. Commonly utilised charge inducers include DCP [9,32] to impart a negative charge, while cetylpyridinium chloride [20] and stearylamine [77] are used to impart a positive charge. The choice of which additive to add usually depends on the payload and target.

### 4.6. Phase Transition Temperature (T_c_)

The phase transition temperature of a surfactant can affect vesicle formation and the ultimate structure of the technology [25]. The inherent phase transition behaviour of surfactants plays an important role in the final properties of dispersion and has an impact on membrane permeability, rigidity of the bilayer, vesicle stability and entrapment efficiency [20]. The phase transition temperature is affected by the length of the alkyl chain of a surfactant and affects the fluidity of the vesicles formed [25]. 

Generally, an increase in the number of carbons in the alkyl chain results in an increase in the gel-to-liquid transition temperature. A higher T_c_ results in vesicles with a lesser propensity to leak the payload [78].

EE is also affected by the T_c_. A higher T_c_ results in higher entrapment efficiency. The mechanism is not well understood but may likely explain the higher entrapment efficiency of colchicine and nimesulide in vesicles formed using Span^®^ 60 [33,70]. Furthermore, flurbiprofen also follows a similar trend when encapsulated in niosomes manufactured using sorbitan esters. The longest chain, Span^®^ 60, exhibits the highest EE [79].

### 4.7. Temperature of Hydration (T_h_)

Hydration temperature influences the size and shape of the vesicles. Ideally, the T_h_ used in niosome manufacture should be higher than the T_c_ of the system. A change in temperature of the system affects the assembly of surfactants into vesicles and may induce vesicle shape transformation [20]. The T_h_ affects the gel–liquid phase of the surfactant and consequently affects the arrangement of the surfactants in the vesicles. For instance, polyhedral vesicles were formed when using a mixture of C_16_G_2_ and Solulan^®^ C_24_ in a ratio of 91:9 at 25 °C, and they transformed into spherical vesicles at 48 °C. However, when cooled from 55 °C, the vesicles produced were a cluster of smaller spherical niosomes at 49 °C prior to forming polyhedral structures at 35 °C. Conversely, vesicles formed using C_16_G_2_, cholesterol and Solulan^®^ C_24_ in a ratio of 49:49:2 revealed no transformation in shape when heated or cooled [74].

## 5. Specialised Niosomes

Niosomes can be formulated with special properties different from conventional or ‘normal’ niosomes to achieve characteristics essential for their use. These modifications are carried out in order to improve the aforementioned characteristics. It should be noted that the manufacture and characterization of specialised niosomes are the same as those for conventional niosomes with the addition of materials that impart some of these specialised functions.

### 5.1. pH-Responsive Niosomes

Niosomes that are pH-responsive are potential drug-delivery systems and are most commonly studied for the targeting of cancer and inflamed tissues due to their acidic nature (pH between 5.5–7.0) in comparison with the normal pH of 7.4 of healthy extracellular tissues [80]. The concept relies on preparing drug-loaded niosomes with pH-sensitive molecules added to the formulation; the niosomes are stable under physiological conditions but undergo structural changes when exposed to pathological conditions [81,82]. These niosomes comprise non-ionic surfactants and cholesterol as basic components; pH sensitivity is achieved through adding molecules such as peptides [83], derivatized lipids [84,85], derivatized surfactants [86] and pH-sensitive polymers [87,88]. 

Pereira et al. [83] formulated pH-sensitive niosomes using the pH (low) insertion peptide (pHLIP) to target cancer cells. Most importantly, pHLIP direct cytoplasmic delivery avoids the trapping of drugs and the delivery system within endosomes and lysosomes, which was a limitation of some of the peptide-based pH-sensitive niosomes. The use of pHLIP in the formulation of nano drug delivery systems has the potential to overcome the selectivity of cancer cells that have either small or lack of enhanced permeation and retention (EPR), leading to improved therapeutic index [83]. This was evident from in vivo results, with a 2–3-fold increase in accumulation and uniform bio-distribution of niosomes in the tumour cells in comparison with non-targeted niosomes. However, there is a paucity of studies; pHLIP-based niosomes can be further explored to deliver a wide variety of anti-neoplastics and drugs for other inflammatory conditions.

Cholesteryl hemisuccinate (CHEMS) is a cholesterol derivative and is one of the common pH-sensitive agents used; it can self-assemble into a bimolecular layer in alkaline media due to its natural lipophilicity and membrane stabilization activity [89]. However, when exposed to an acidic environment, it breaks away from its bimolecular layer, resulting in the rupture of the bilayer and release of the payload [89].

Niosomes of Tanshinone IIA (TanIIA), a highly lipophilic active constituent with a wide range of anti-tumour activity, have been prepared to improve anti-hepatocellular carcinoma effects. In vitro dissolution studies of niosomes prepared using CHEMS showed sustained drug release for more than 12 h, slightly faster release at pH 6.81, and significantly faster release than that of plain niosomes at pH 5.60 [84]. 

Cisplatin (Cis) niosomes developed using CHEMS as a bilayer stabilizing agent also exhibited sustained drug release for 24 h, and the rate of drug release was pH dependent, with greater drug release at pH 5.4 [85]. An MTT assay of free and cis-loaded niosomes significantly decreased the number of viable MCF7 cells (*p* < 0.05), where the cis-loaded niosomes were more cytotoxic than free drug at all time intervals and concentrations studied, which could be due to better targeting of the drug by the niosomes. The authors also acknowledged the importance of further studies to determine the exact mechanism involved in this phenomenon [85].

Barani et al. [87] utilized CHEMS to develop paclitaxel-loaded pH-responsive niosomes and studied their in vitro and in vivo anti-cancer effects. The study found similarities with results obtained by previous studies in terms of particle size distribution, in vitro drug release patterns and cell culture studies, with lower IC_50_ values reported for pH-sensitive niosomes than those of free drug. The study also confirmed stability for up to 3 months when stored at 4 °C [87].

Ammonium glycyrrhizinate-loaded niosomes (AGN) have been explored as a potential nanotherapeutic system for anti-inflammatory activity in murine models [90]. The study demonstrated that neutral or pH-sensitive AGN are desirable nanotherapeutics for anti-inflammatory and antinociceptive therapy [90].

CHEMS has further been utilized as a pH-sensitivity-imparting agent in the development of treatment for melanoma [91]. Interestingly the researchers further explored the use of oleic acid as a pH-sensitive agent in the formulation, and reported the success of both technologies while highlighting that oleic acid could be successfully utilized for this purpose [91].

Niosomes manufactured from Span^®^ 60 and Tween^®^ 20 have been manufactured and stabilized by either cholesterol or CHEMS and loaded with ibuprofen [92]. The influence of vesicle composition on skin accumulation and transdermal permeation of the payload across excised hairless rat skin was investigated. Niosomes composed of Span^®^ 60 and CHEMS had a statistically significant increase in skin permeation of payload [92].

A novel Tween^®^ 20–glycine derivative surfactant was used as a pH-sensitive material in the manufacture of niosomes [93]. The novel surfactant derivatives were developed for use in hepatoblastoma cells.

The aforementioned novel Tween^®^ 20–glycine surfactant has further been utilized in the development of ibuprofen niosomes, where the bilayer of the niosomes was destabilized upon exposure to acidic pH, resulting in payload release [86]. Niosomes administered at the site of inflammation via subcutaneous (SC) injection in CD-1 male mice showed a significant pain threshold between 2 to 4 h in comparison with plain niosomes, which exhibited only 1 h, indicating their potential for targeted drug delivery of lipophilic drugs. In addition, the study confirmed the stability of the niosomes for up to 3 months when stored at 4 °C [86].

### 5.2. Magnetic Niosomes

**Figure 4:**
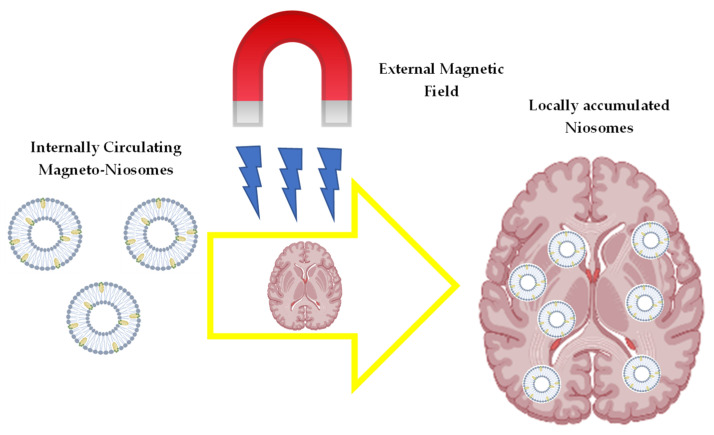
Schematic representation of magnetized niosome accumulation in targeted drug delivery.

Niosomes have the potential for use in combining drug delivery and magnetic targeting for cancer therapy and diagnosis [94,95]. The basic concept of using magnetic materials is to permit the targeting of drug-loaded magneto-niosomes to specific organs or tissues through the application of extracorporeal magnets [94]. A schematic representation of magneto-niosomes and their localisation is depicted in Figure 4.

Doxorubicin-loaded magneto-niosomal formulations have successfully been manufactured with the magnetic material EMG 707 ferrofluid encapsulated in the aqueous core of the niosome. The niosomes exhibited controlled release of the payload with few toxicity concerns [96]. Davarpanah et al. [97] successfully developed PEGylated magnetic niosomes loaded with carboplatin using film hydration. The PEGylated magnetic niosomes showed very good entrapment efficacy of the drug at 83% and had a mean diameter of 145 nm. PEGylation of the magnetic niosomes improved bioavailability and resulted in the sustained release of the drug. The improved bioavailability was confirmed through the viability of the MCF-7 cancer cell line when exposed to the magnetized niosomes with (38%) and without (57%) the application of an external magnetic field. 

Barani et al. [98] studied the potential of magnetic niosomes for the delivery of genes into the cytoplasm and nucleus. As reported by the authors, the study was the first of its kind and revealed better bilayer stability of lipid membranes for ergosterol niosomes over cholesterol due to the high number of hydrogen bonds between ergosterol and the surfactants. This was essential for the delivery of large molecules. In terms of entrapment efficacy, the niosomes could load up to 30% of the plasmid, with a mean size of 70 nm. The transfection efficacy of the plasmid was 1.4-fold higher when a magnetic field was applied. Additionally, the ergosterol-based niosomes were found to be biocompatible due to lower cytotoxicity observed when exposed to HEK-293T cells.

Maurer et al. [99] used a similar approach to downregulate the LFG gene in BT-474 breast cancer cells by delivering small interfering RNA (siRNA) using superparamagnetic iron-oxide nanoparticle-based niosomes. Application of an external magnetic field improved the internalization of siRNA, resulting in efficient apoptosis. These studies form a very good basis for further research in transferring genes into cancerous cells to promote death or to slow the growth and improve the efficacy of other chemotherapeutic agents at reduced doses [99].

Jamshidifar et al. designed and developed magnetic niosomal nanocarriers for co-delivery of curcumin and letrozole into breast cancer cells. The magnetic core was NiCoFe_2_O_4_ coated by a thin layer of silica, which was then encapsulated in a niosome, allowing co-loading of letrozole and curcumin into the silica-containing layer and hydrophobic bilayer, respectively [100]. Cellular assays revealed the niosomes’ specificity for cancer cells, exhibiting low cellular uptake in MCF-10A non-tumorigenic cells, and related high viability, but high cellular uptake in MDAMB-231 and SK-BR-3 cancer cells. The cytotoxicity profile of the co-loaded niosomes was superior to that of the aqueous solutions of both drugs, indicating their enhanced cellular uptake in their encapsulated states. In particular, the magnetic co-loaded niosomes showed the greatest cytotoxicity effects on MDA-MB-231 and SK-BR-3 breast cancer cells. The observed cytotoxicity was attributed to regulation of the expression levels of the genes in breast cancer cells [100].

### 5.3. Immuno-Niosomes

Niosomes have been conjugated with antibodies to form immuno-niosomes, and conjugation of monoclonal IgG antibodies to vesicle surfaces was achieved by incorporation of cyanuric chloride derivatisation of Tween^®^ 61 [101].

PEGylation of immune-niosomes has been reported to result in better binding efficacy and the protection of proteins used to elicit the immunogenic response [100].

Hood and co-workers used the anti-CD44 antibody IM7 linked via a cyanuric chloride linkage and targeted fixed cells known to express CD44 to deliver anti-inflammatory agents [101]. The manufactured niosomes showed selectivity and specificity in comparison to controls. These findings suggest that the resulting immuno-niosomes may provide an effective method for targeted drug delivery [101].

Similarly, niosomes have been manufactured to target CD44+ receptors to treat atherosclerosis [102]. Static binding data suggested that for short periods, i.e., 5, 10, 15 and 30 min, niosomes containing 5% PEG and those containing 10% Tween^®^ 61 exhibited similar binding characteristics. However, at longer periods, i.e., 60 and 120 min, the niosomes containing 5% PEG displayed improved binding compared to the other niosomes [102]. The targeting potential of the niosomes was achieved by functionalizing them with an anti-CD44+ antibody (KM-201), while binding potential and circulation duration were improved via PEGylation [102].

A study conducted by Gogoi et al. [103] reported the protective efficacy (80%) of the niosomal formulation of a protective antigen (PA) that is responsible for eliciting neutralizing antibodies against anthrax. The niosomal formulation of PA showed better protection against *Bacillus anthracis* infection as compared to only PA. An in vitro release assay showed a bi-phasic approach with an initial burst of the antigen for 24 h followed by sustained release, indicating the potential of the niosomes as an adjuvant for prolonged immune response, which can further assist with reducing the requirements of multiple booster vaccines [103].

### 5.4. Thermoresponsive Niosomes

Thermoresponsive niosomes are another type of stimuli-responsive niosome that are studied for targeted drug delivery to tumour cells. These systems operate through the process of accumulating in the solid tumour due to the EPR effect and release the drug when the target tissue is subjected to mild hypothermia through the application of heat locally. The application of heat results in phase transition (T_c_) of the non-ionic surfactant membrane from a gel-to-liquid state, resulting in destabilization of the bilayer and hence release of the drug [104]. Thermal triggering can be achieved through the inclusion of a thermo-sensitive surfactant, polymer or peptide with a transitional temperature between 38 to 42 °C in the niosomal formulation. 

**Figure 5:**
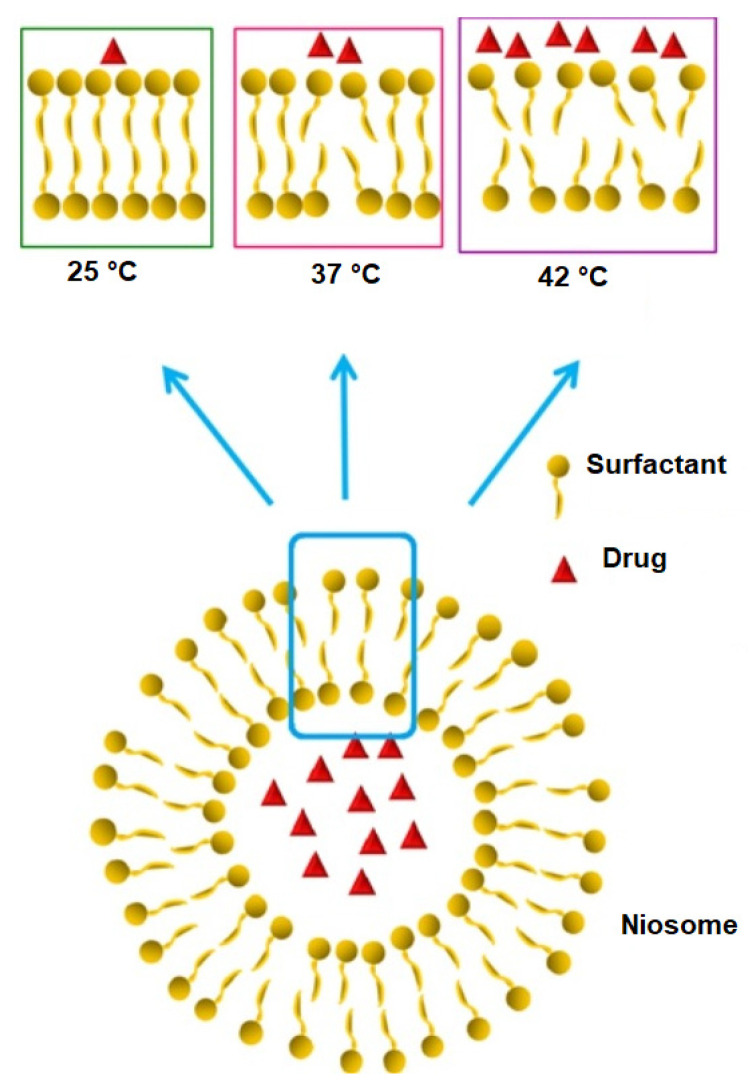
Schematic representation of temperature effect on payload release from thermoresponsive niosomes. (Reproduced from [105] with permission from Elsevier, Amsterdam, 2022.).

Tavano et al. [105] first reported the use of a thermo-sensitive polymeric surfactant in the preparation of thermoresponsive niosomes. Pluronic^®^ L64 and L64ox, a derivative of L64, were used as polymeric surfactants, and niosomes were prepared with or without cholesterol using film hydration. Multilamellar niosomal vesicles formed and showed good stability (>4 weeks), good EE (~85%) and nano-size range (335–600 nm). In vitro drug release studies found that only L64 niosomes without cholesterol met the thermo-responsive release criteria, where at 25 °C niosomes existed as stable bilayers, at 37 °C, surfactant chain mobility increased, and membrane destabilization at 42 °C led to a higher drug release rate, as depicted in Figure 5. The authors found similar release patterns for the drugs calcein and 5-fluorouracil, indicating that the phenomenon is more dependent on the surfactant than the loaded drug. 

A very recent study by Damera and Nag [106] worked on preparing hybrid thermo-responsive niosomes using Span^®^ 60, Pluronic^®^ L64 and cholesterol. Data from steady-state fluorescence anisotropy and Raman spectroscopy showed a phase transition temperature of 35 to 36 °C for niosomes of L64, which can be correlated with the findings of Tavano et al. [105]. However, the addition of Span^®^ 60 to Pluronic^®^ L64 (80:20) shifted the transition temperature to 40 °C, which decreased with increased concentration of Span 60 to below the average body temperature of 37 °C, making it not meet the criteria mentioned above.

Despite the dearth of work performed on temperature-triggered niosomes, formulation strategies such as the use of thermo-responsive peptides (elastin-like polypeptide (ELP)) [107] and polymers [104] in the successful preparation of liposomes can be extrapolated to expand the applications of these niosomes. To note, both studies lack data for in vivo safety and efficacy, which can be an area for future exploration. 

### 5.5. Stealth Niosomes

**Figure 6:**
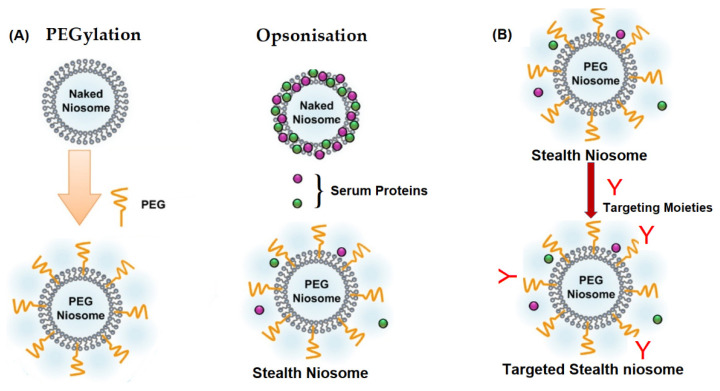
Schematic representation of (**A**) naked and PEGylated niosome interactions with serum proteins and (**B**) targeted stealth niosomes. (Adapted from [108] with permission from Elsevier, Amsterdam 2022).

Stealth, or long-circulating, niosomes are developed to overcome rapid removal from blood circulation due to uptake by the mononuclear phagocytic system (MPS), resulting in clearance before reaching the target site [107]. In recent years, PEGylation of nanoparticles has been reported extensively to achieve stealth properties. PEG chains protect the nanoparticles from MPS by creating a hydrophilic film on the surface, which repels serum protein interactions responsible for opsonization (Figure 6A) [107,108]. Further addition of targeting moieties such as monoclonal antibodies (Mab), peptides, growth factors, etc., (Figure 6B) result in the accumulation of drug in target tissues, resulting in selective therapeutic activity [109].

Earlier studies for preparing stealth niosomes reported the use of Poly (methoxypolyethyleneglycol cyanoacrylate-co-n-hexadecyl cyanoacrylate) (PEG-PHDCA). PEGs of various molecular weights have been studied (2000, 5000 and 10,000), showing a significant reduction (~1.69%) in phagocytic uptake for PEG 5000-PHDCA niosomes in comparison with PHDCA niosomes (~14.4%), indicating the stealth effects of the copolymer [109]. The PS of the PEG 5000-PHDCA niosomes also played a significant effect on phagocytic uptake, with less uptake observed for smaller niosomes. In vitro drug release studies showed an inverse relation with PEG chain length, which was attributed to the loose arrangement of the polymer with increased chain length. In vivo blood clearance studies showed the presence of PEG-PHDCA niosomes even after 24 h, indicating the long circulation ability, which could be further confirmed through various pharmacokinetic parameters, i.e., t_1/2_ and AUC [109]. 

Shehata et al. [108] reported the in vivo efficacy of PEGylated niosomes of various non-ionic surfactants: Brij^®^ 72, Span^®^ 20 and Tween^®^ 60. Surface coating with PEG resulted in reduced PDI and a ZP towards neutral, indicating the formation of monodispersed niosomes and stearic hindrance. Serum protein interaction studies indicated better performance for Brij^®^ 72-PEG niosomes with regards to decreased drug release, while no significant difference was observed for Tween^®^ 60. The authors attributed this phenomenon to the number of polyoxyethylene chains, where Tween^®^ 60 acted as PEG; hence, no significant difference was observed in comparison with the naked. In vivo pharmacokinetic studies indicated a significant difference in the clearance time between naked and PEGylated for Brij^®^ 72 and Span^®^ 20, indicating the potential for these surfactants in the preparation of stealth niosomes.

Similarly, Haroun and co-workers [110] reported the use of Brij^®^ 52, Span^®^ 60 and Poloxamer 184 surfactants in the preparation of stealth niosomes. Results of the study indicated PEGylation of these niosomes as potential targeted drug delivery systems. The study reported superior performance for Span^®^ 60-PEGylated niosomes for serum protein, resulting in fewer interactions in in vitro release studies conducted in both buffer and serum and in in vivo anti-tumour activity [110].

Starting with the delivery of nucleic acids, DNA vectors and oligonucleotides all the way to the latest RNA interference (RNAi), microRNAs (miRNAs), short interfering double-stranded RNAs (siRNAs) and CRISPR-Cas9, these methods need suitable drug delivery systems. Several cationic niosomes have been reported in the literature for their potential to act as vectors in gene therapy [111]. However, one of the stumbling blocks for effective drug delivery with these systems is non-specific plasma protein interactions, where cationic vessels were reported to bind with proteins with an isoelectric point (pI) < 5.5 [112]. This could have a negative impact on transfection efficiency, which affects therapeutic outcomes.

Pengnam et al. [113] reported the use of PEGylated niosomes to overcome these challenges—PEGylated plier-like cationic niosomes containing Span^®^ 20 and cholesterol for gene delivery were studied. The resultant cationic nioplexes showed a positive zeta potential that decreased with an increase in the amount of PEG added. The transfection efficiency was significant in niosomes with 2% of PEG (118%) in comparison to the normal cationic niosomes. However, this was achieved at a weight ratio of 10 in comparison to that of the normal cationic niosomes of 2.5. The study further confirmed the protection of pDNA from serum nuclease for at least 6 h by the PEGylated niosomes [113].

Immordino et al. [111] discussed PEG alternates in the formulation of stealth liposomes. Polymers such as polyvinyl alcohol, polyvinyl pyrrolidone, polyacryl amide, poly[N-(2-hydroxypropyl) methacrylamide], amphiphilic poly-N-vinylpyrrolidones, etc., with similar or better hydrophilic nature, flexible main chains and biocompatibility were reported to exhibit prolonged circulation time, indicating their potential for stealth formulations. However, not many of these polymers were studied in the formulation of stealth niosomes, which is noteworthy. Further, the potential for stealth niosomes for prolonged blood circulation, leading to increased area under the curve and selective tissue distribution through targeted drug delivery, makes them attractive systems for the treatment of various diseases, such as cancer.

### 5.6. Deformable Vesicles (Transfersomes and Ethosomes) 

**Figure 7:**
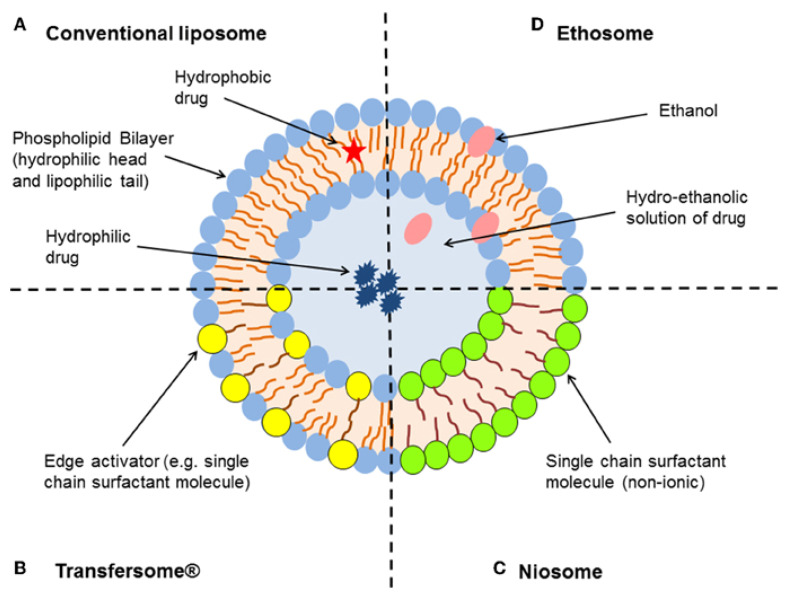
Comparative pictorial representation of conventional liposome (**A**), transfersome (**B**), niosome (**C**) and ethosome (**D**). (Reproduced with permission from [117] and Frontiers in accordance with the Creative Commons Attribution License).

Transferosomes and ethosomes are unique, ultra-deformable and self-optimized aggregate systems with combined characteristics of both liposomes and niosomes, as depicted in Figure 7. They consist of a large concentration of lipids (70% and above), and edge activators (EA) play a very important role in modulating fluidity and elasticity, thus enhancing the capabilities of conventional liposomes and niosomes to reach deeper layers of the skin [114]. Due to their capability to penetrate the pores of the stratum corneum by either intracellular or transcellular routes, transferosomes have been proven to be highly promising non-invasive transdermal drug delivery systems (TDDS) for transporting both lipophilic and hydrophilic drugs, drugs with large molecular weight (MW) up to 1 kDa and macromolecules such as peptides or proteins [115,116]. 

The primary composition of transferosomes includes the amphipathic vesicle forming agent such as phospholipids (phosphatidylcholine), 10–25% surfactants/EA (sodium cholate, sodium deoxycholate, dipotassium glycyrrhizinate, Tweens^®^, Spans^®^, etc.), 3–10% penetration enhancer (ethanol or methanol) and the hydrating medium (water or a saline phosphate buffer with pH 6.5–7) to promote osmotic force and facilitate permeation [114,115]. Factors such as phospholipids: EA, type of EA, solvent and pH of the hydration medium used in manufacturing play critical roles in the optimisation process. These factors affect the PDI, EE, elasticity (phospholipids: EA and type of EA), permeation across stratum corneum (solvent, type and concentration of EA, carbon chain length and transition temperature of EA) and ionization of the drug (pH of the hydration medium) [115,118].

Transferosomes have been extensively studied for their application in the transdermal delivery of various therapeutic agents, including vitamins, supplements, corticosteroids, local anaesthetics, anti-inflammatories, antifungals, antioxidants, antineoplastics, antipsychotics, antihypertensives, antihistamines, antibiotics, anti-amoebics, anti-diabetics; macromolecules such as Interleukin-2, Interferon-α, insulin, etc.; hormones such as estradiol; and vaccines such as tetanus-toxoid. Studies conducted with these agents indicated the potential of transferosomes for increased permeability, sustained drug release leading to prolonged therapeutic efficacy, improved transdermal flux, reduced blood clearance and enhanced pharmacokinetic profile [114,115,119,120,121,122]. As there are many comprehensive reviews presented in the literature for the aforementioned therapeutic classes of drugs [116,123], the following sections will illustrate additional and exciting applications of deformable vesicles.

**Figure 8:**
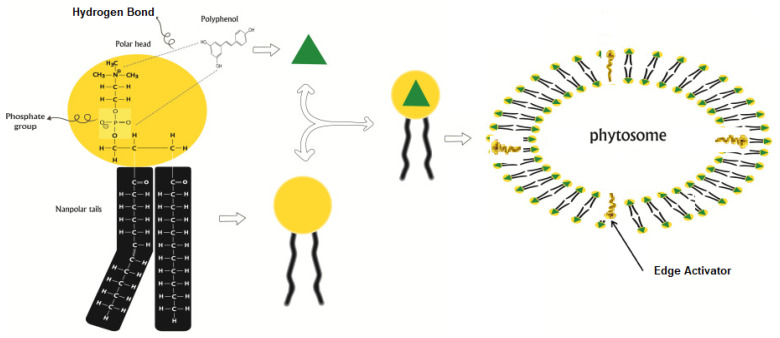
Pictorial representation of phytosome formation. (Adopted from [121] with permission from Frontiers in accordance with the Creative Commons Attribution License).

Delivery of phytochemicals using transferosomes (phytosomes) has been explored to improve the therapeutic efficacy of various phytochemicals with low absorption rates across the skin. As depicted in Figure 8, phytosomes are formed through phospholipid interaction with phytochemicals via the hydrogen bond between the polar functions of the phospholipid and the phytochemical constituents. Permeation of these phytosomes through the skin is achieved by the addition of EA into the phospholipid bilayers [121,124]. The majority of research has been done to improve the permeability of polyphenolic compounds such as flavonoids for improved bioavailability. Studies have been carried out to evaluate the biological activities of phytochemicals for anti-ageing, antihypertension, neurological, anti-inflammatory, antioxidant, antineoplastic and antibiotic properties, with noteworthy commercial success [121,124,125]. As new phytochemicals are discovered at a rapid pace, much research should be undertaken to overcome the limitations of these pharmaceutical and nutraceutical applications.

Deformable vesicles have different mechanisms of penetration in comparison to niosomes and liposomes and tend to locate in the more-hydrated layers of skin tissues and are therefore more efficient for transdermal drug delivery [19,126,127,128].

Transferosomes of papaverine hydrochloride (PH) have been studied for their potential in the treatment of erectile dysfunction (ED) as an alternative to painful and invasive intracavernosal injections and intraurethral therapy. The optimal EE was found to be at 73% with a particle size of 200 nm. However, these were found to be unstable as storage temperature increased, leading to increased vesicle size and reduced EE to 38% at 37 °C. However, loading the transferosomes into a gel system substantially improved stability for 30 days at 25 °C [122].

Nanosized transferosomes of olanzapine (OLZ) have been prepared. Intranasal administration of OLZ in Wister albino rats showed no signs of necrosis or haemorrhage after 14 days [129]. The pharmacokinetic profile indicated a three-fold higher drug concentration when administered intravenously as compared with the transferosomes. Low concentration levels in the brain were attributed to rapid nasal clearance in rats, the interaction of the vesicle membrane with serum proteins and destabilisation of vesicle membrane lipid contents. The study also found that the flexibility of the transferosomes played a significant role in improving drug delivery to the brain [129].

Mazyed and Abdelaziz [130] studied transferosomes for ocular drug delivery. Transferosomes of acetazolamide (ACZ) were formulated and loaded into a gel system made up of poloxamers. Drug-release studies after 8 h showed prolonged drug release and higher ex vivo permeation for gel-based transferosomes, with a significantly lower intraocular pressure for 24 h [130].

The application of hydrogel-based nanocarriers for nose-to-brain and eye-to-brain drug delivery is currently state-of-the-art for addressing CNS conditions. Knowing the stability issues of deformable vesicles due to oxidation, using a gel system seems to be promising. The combined advantages of transferosomes and gel systems along with the addition of molecules that can provide functional properties such as stimuli–response could be an area of further research to improve delivery of drugs and gene-modifiers to the CNS.

### 5.7. Radio Niosomes

Much like any other payload, radiopharmaceuticals have been delivered using niosomes. Niosomes loaded with radiopharmaceuticals have potential uses in many cancers. 

These fabricated radio-labelled niosomes have a critical use in diagnostic imaging of organs such as the liver and spleen. For instance, ^99m^Tc-labelled diethylenetriamine pentaacetate (DTPA) has demonstrated potential use for imaging [131].

Similarly, Munekane and co-workers manufactured (PEGylated) Span^®^ 20 niosomes and labelled them with indium-111 (I-111) using a remote-loading method [132]. The novel radio-niosome formulation was assessed in vivo and compared to that of a liposome. In addition, niosomes based on Spans^®^ 20, 40, 60 and 80 were compared to evaluate the effect of the lipophilicity of the surfactants on niosome formulation characteristics. The manufactured PEGylated niosomes exhibited >95% labelling efficiency and purity. The stability of ^111^In-labeled niosomes in the serum was high enough to trace in vivo behaviour. There was a significant extension in retention of Span^®^ 20 niosomes in the blood and high accumulation in the tumour 48 h post-injection when compared to the liposome. Niosomes manufactured from Span^®^ 80 with an unsaturated hydrocarbon chain exhibited decreased radioactivity in the blood and increased accumulation in the spleen compared to niosomes manufactured from Spans^®^ 20, 40 and 60 that contained saturated hydrocarbon chains. It is worth noting that all the manufactured niosome formulations exhibited high accumulation in the tumour, suggesting that radiolabelled and PEGylated niosomes can be useful as tumour-targeting drug carriers [132]. 

De Silva et al. successfully demonstrated the manufacture of prototypical ^99m^Tc conjugated niosomes through a simple and fast one-step method [133]. Their work indicated the feasibility of the developed radio-niosomes for in vivo administration, resulting in high tumour to muscle uptake.

Overall, radio-niosomes have the potential to improve the performance of niosomes in imaging and could find a further place in combination with magneto-niosomes.

## 6. Limitations 

Despite their flexibility in routes of administration, type of payload to encapsulate and ability to sustain the release of payload systematically, niosomal technology is not without drawbacks.

The main concern regarding the development of niosomal formulations lies in the sterilization process to be utilised. Heat sterilization using dry heat and steam sterilization are inappropriate as they are destructive to lipid or surfactant-based formulations with a T_c_ lower than the temperature used in the sterilization process. As such, use of these techniques would result in extreme drug leakage as a consequence of bilayer collapse [5]. By the same token, membrane filtration would not be ideal for niosomal formulations with a PS greater than the 220 nm pore size of the membrane filters. A possible solution to circumvent this would be to prepare niosomes under aseptic conditions or to use sterilization techniques that make use of minimal heat-generating radiation [21,134].

Further concerns associated with niosomes lie in their stabilization while in dispersion. The composition of niosomes is such that additives have to be included in the formulation to improve long-term physical stability. This phenomenon was demonstrated during long-term stability assessments of nevirapine niosomes in which niosomes that had DCP included in the formulation exhibited superior stability when compared to niosomes formulated without a charge-inducing agent [9,10]. While the components of niosomes are generally regarded as safe (GRAS), there are still some concerns regarding the safety of niosomal formulations. Despite the dearth of studies regarding the toxicity of niosomes, there is potential for surfactants to provide some degree of toxicity based on their segregation. In one study, the inhibition of human keratinocyte cell proliferation by different niosomal formulations, particularly the effects of surfactants and concentration of cholesterol, was evaluated [135,136]. The study examined surfactants of different hydrocarbon chain lengths and polyoxyethylene chain lengths. Furthermore, ether- and ester-type surfactants were also investigated. The data generated revealed that both the hydrocarbon chain and the polyoxyethylene chain length had minor effects on cell proliferation. However, the bond by which the alkyl chain was linked to the polyoxyethylene head group had a significant influence on cell proliferation. Ester-type surfactants exhibited lower toxicity compared to ether-type surfactants, which was attributed to enzymatic degradation of the ester bond. Moreover, the addition of cholesterol as a bilayer stabilizer had no effect on cell proliferation [135,136].

The toxicity of niosomes in ocular applications has recently been investigated by measuring the conjunctival and corneal irritation potential of Span^®^ 60 niosomes and surface-modified Span^®^ 60 niosomes using hen’s egg chorioallantoic membranes and excised bovine corneal opacity and permeability models. Niosomes exhibited minimal ocular irritation, which suggests good ocular tolerability [137]. It needs to be noted that data related to the cytotoxicity of niosomal formulations as well as the individual components viz., surfactant molecules, cholesterol and charge inducers have been reported by many papers. Nevertheless, there have been no specific studies aimed at investigating the toxicity of niosomes post administration in animal models. There is a need to perform these long-term studies in animal models to assess the possibility of translating this technology to clinical applications.

## 7. Prospects and Conclusions

Recently, niosomes have been extensively studied for various applications, from topical, transdermal and oral to brain-targeted drug delivery [21]. Their production is relatively easy and cost-effective, while they are capable of achieving higher EE than their predecessors, liposomes. This versatile technology has great potential in the fields of pharmaceutical, veterinary and cosmetic sciences due to their potential enhancement by novel preparation techniques, modification methods to tailor delivery and novel formulation components, which would enable them to achieve targeted delivery, better drug entrapment efficiency and to develop specialised niosomes with special structures. Furthermore, specialised niosomes can be combined with stimuli-responsive carrier gels and/or eutectic/ionic liquids to increase their residence times at sites of action.

There is a dearth of work exploring the possibility of utilizing amphiphiles that possess biological activity or that can be used as targetable ligands as the core component of niosomes. Despite this, some materials with a duality of functions have been synthesized. For instance, Uchegbu et al. synthesized palmitoyl muramic acid and N-palmitoyl glucosamine and manufactured niosomes from these materials [138]. This approach could be extended to the manufacture of surfactants that have the ability to self-assemble and have biological effects, potentially imparting some of the aforementioned specialised niosomal functions that would further improve the functionality of the novel niosomes.

The use of specialised niosomes with improved performance compared to conventional niosomes appears to be one of the most effective ways to further derive utility from this technology and to potentially drive innovation toward developing market-ready products.

## Data Availability

Not applicable.
